# Effect of density dependence on coinfection dynamics

**DOI:** 10.1007/s13324-021-00570-9

**Published:** 2021-09-21

**Authors:** Jonathan Andersson, Samia Ghersheen, Vladimir Kozlov, Vladimir G. Tkachev, Uno Wennergren

**Affiliations:** 1 Department of Mathematics, Linköping University, Linköping, Sweden; 2 Department of Physics, Chemistry, and Biology, Linköping University, Linköping, Sweden

**Keywords:** SIR model, Coinfection, Carrying capacity, Global stability

## Abstract

In this paper we develop a compartmental model of SIR type (the abbreviation refers to the number of Susceptible, Infected and Recovered people) that models the population dynamics of two diseases that can coinfect. We discuss how the underlying dynamics depends on the carrying capacity *K*: from a simple dynamics to a more complex. This can also help in understanding the appearance of more complicated dynamics, for example, chaos and periodic oscillations, for large values of *K*. It is also presented that pathogens can invade in population and their invasion depends on the carrying capacity *K* which shows that the progression of disease in population depends on carrying capacity. More specifically, we establish all possible scenarios (the so-called transition diagrams) describing an evolution of an (always unique) locally stable equilibrium state (with only non-negative compartments) for fixed fundamental parameters (density independent transmission and vital rates) as a function of the carrying capacity *K*. An important implication of our results is the following important observation. Note that one can regard the value of *K* as the natural ‘size’ (the capacity) of a habitat. From this point of view, an isolation of individuals (the strategy which showed its efficiency for COVID-19 in various countries) into smaller resp. larger groups can be modelled by smaller resp. bigger values of *K*. Then we conclude that the infection dynamics becomes more complex for larger groups, as it fairly maybe expected for values of the reproduction number $R_0\approx 1$. We show even more, that for the values $R_0>1$ there are several (in fact four different) distinguished scenarios where the infection complexity (the number of nonzero infected classes) arises with growing *K*. Our approach is based on a bifurcation analysis which allows to generalize considerably the previous Lotka-Volterra model considered previously in Ghersheen et al. (Math Meth Appl Sci 42(8), 2019).

## Introduction

Two or more pathogens circulating in the same population of hosts can interact in various ways. One disease can, for instance, impart cross-immunity to the other, meaning that an individual infected with the first disease becomes partially or fully immune to infection with the second [7, 18]. One disease can also mediate the progression of another disease in a population.

Therefore it is important to understand the dynamics of coexistent pathogens. In epidemiology the interaction of strains of the same pathogen, such as influenza or interacting diseases such as HIV/AIDS and hepatitis is very common and involves many complexities. The central problem in studying such systems is the explosive growth in the number of state variables of the system with the linear increase in the number of strains or pathogens [13]. Mostly these strains or pathogens are interacting in a way which has limited the analytical progress in understanding the dynamics for such systems. In this regard, it is a challenge to understand the dynamics and evolution of pathogens in populations. The complexity of multiple strain models allows a great variability in modelling strategies. Recently, attention has focused on understanding the mechanisms that lead to coexistence, competitive exclusion and co-evolution of pathogen strains in infectious diseases which is important from the management of disease perspective.

Several studies exist on the coinfection with specific diseases. There is also an active research [7, 14, 16, 17, 19] which has addressed this issue in general. In [6], a mathematical model has been studied and it showed that for strains with differing degree of infectivity, all strains will get extinct except those that have the highest basic reproduction number. Allen et al in [1] showed coexistence only occur when the basic reproduction number is large enough for persistence of strains. They numerically illustrate the existence of globally stable coexistence equilibrium point. In another study, Allen et al [2], studied an SI model of coinfection with application on hanta virus. They assumed a logistic growth with carrying capacity and horizontal transmission of both viruses and yet only vertical transmission of virus 2. The condition of coexistence of two strain is described.

In [4], a SIR model with vertical and horizontal transmission and a different population dynamics with limited immunity is considered. It is shown that the competitive exclusion can occur which is independent of basic reproduction number but a threshold. The existence and stability of endemic equilibrium is also shown. Since coinfection involves many complexities, many studies are only restricted to numerical simulations to understand the dynamics.

Nevertheless, mathematical modelling is one of the effective tool to understand the dynamics of biological system. But the major challenge is to balance between the practicality and mathematical solvability of the model. The cost of realisticity in mathematical modelling is the diminution of mathematical machinery.

The way to deal with this challenge is to divide the model into different sub models. The differences between the models is due to different biological assumptions. There are two major advantages with this approach. First is the understanding of the system completely under certain assumptions. It can help to apply it to some real-life situations, since the controlling strategies for a diseases sometimes transforms the original system to a more simple one. In those cases the complete information about such simplified system is needed to deal with that type of unexpected situation from management prospective. The second is, by relaxing assumptions, one can understand the role of each new parameter and its effects on the dynamics of epidemic.

One of the important characteristics, to understand the coinfection dynamics is transmission mechanism. In paper [12] we have developed a SIR model to understand the dynamics of coinfection. Limited transmission is considered and the competitive exclusion principle is observed. The transition dynamics is also observed when the equilibrium points exist in the form of branches for each set of parameters. The complete dynamics of the system for all set of parameters is described by using linear complementarity problem. It appeared that there always exist an equilibrium point which is globally stable. It is showed that the dynamics of the system changes when carrying capacity changes. There are certain assumptions on the transmission of coinfection in that model. It is assumed that the coinfection can only occur as a result of contact between coinfected class and susceptible class, coinfected class and single infected classes. Interaction between two single infected classes is not considered. Also the simultaneous transmission of two pathogens from coinfected individual to susceptible individual is assumed.

In this paper we develop a density dependent SIR model for coinfection which is a relevant extension of the model presented in [12] to understand the role of each new transmission parameter in the dynamics. Our aim here is to investigate how the dynamics changes due to a certain parameter, which in our case is the carrying capacity *K*, from a simple dynamics to a more complicated. This can help in understanding the appearance of more complicated dynamics for example chaos etc. Contrary to [12], we could no more make use of the linear complementarity problem due to some additional term which appeared by relaxing the assumption of interaction between two single infected classes. We instead used a technique based on bifurcation analysis. The density dependent population growth is also considered. It is presented that pathogens can invade in population and how their invasion depends on the carrying capacity *K*.

**Fig. 1:**
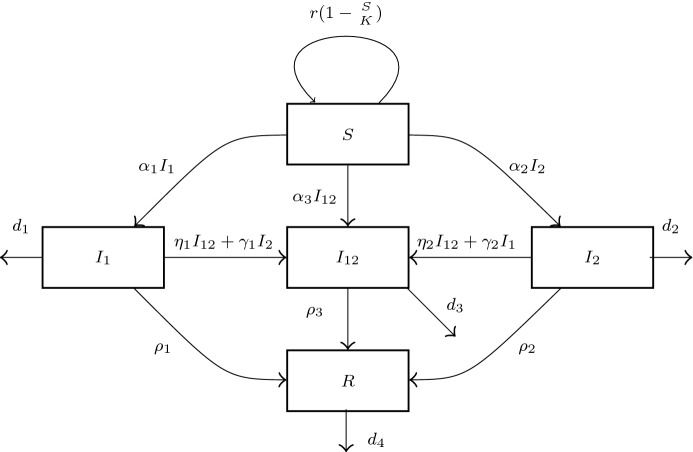
Flow diagram for two strains coinfection model. The expression next to the arrows indicates the relative flow out of the respective compartment

##  Model formulation and the main result

### The model

The present model is displayed in Fig. 1. More precisely, we assume that the single infection cannot be transmitted by the contact with a coinfected person. According to Fig. 1, this process gives rise to the system of ODEs:

1 $$\begin{aligned} \left\{ \begin{array}{ll} S' &{}=(r(1-\frac{S}{K})-\alpha _1I_1-\alpha _2I_2-\alpha _3I_{12})S,\\ I_1' &{}=(\alpha _1S - \eta _1I_{12}-\gamma _1I_2 - \mu _1)I_1,\\ I_2' &{}=(\alpha _2S - \eta _2I_{12}-\gamma _2I_1- \mu _2)I_2,\\ I_{12}' &{}=(\alpha _3S+ \eta _1I_1+\eta _2I_2-\mu _3)I_{12}+\overline{\gamma }I_1I_2, \\ R' &{}=\rho _1 I_1+\rho _2I_2+\rho _3 I_{12}-d_4 R, \end{array} \right. \end{aligned}$$

where we use the following notation:

- *S* represents the susceptible class,
- $I_1$ and $I_2$ are the infected classes from strain 1 and strain 2 respectively,
- $$I_{12} $$ represents the co-infected class,
- *R* represents the recovered class.

Following [2, 6, 20], we assume a limited population growth by making the per capita reproduction rate depend on the density of population. The recovery of each infected class is presented by the last equation in (1). The fundamental parameters of the system are:

- $r=b-d_0$ is the intrinsic rate of natural increase, where *b* is the birthrate and $d_0$ is the death rate of *S*-class,
- *K* is the carrying capacity (see also the next section),
- $\rho _i$ is the recovery rate from each infected class ($i=1,2,3$),
- $$d_i $$ is the death rate of each class, $$( i=1,2,3,4)$$, where $$d_3$$ and $$d_4$$ correspond $$I_{12}$$ and *R* respectively,
- $$\mu _i=\rho _i+d_i, i=1,2,3.$$
- $\alpha _1$, $\alpha _2$, $\alpha _3$ are the rates of transmission of strain 1, strain 2 and both strains (in the case of coinfection),
- $\gamma _i$ is the rate at which infected with one strain get infected with the other strain and move to a coinfected class ($i=1,2$),
- $${\bar{\gamma }}=\gamma _1+\gamma _2$$,
- $\eta _i$ is the rate at which infected from one strain getting infection from a co-infected class $( i=1,2)$;

Summing up all equations in (1) we have

2 $$\begin{aligned} \begin{array}{l} N'=r(1-\frac{S}{K})S-d_1I_1-d_2I_2-d_3I_{12}-d_4 R \end{array} \end{aligned}$$

where $N=S+I_1+I_2+I_{12}+R$ is the total population.

We only need to consider the first four equations of (1) since *R* appears only in the last equation, hence it does not affect the disease dynamics. Rewrite the reduced system as

3 $$\begin{aligned} \left\{ \begin{array}{ll} S' &{}=(r(1-\frac{S}{K})-\alpha _1I_1-\alpha _2I_2-\alpha _3I_{12})S \\ I_1' &{}=(\alpha _1S - \eta _1I_{12}-\gamma _1I_2 - \mu _1)I_1 \\ I_2' &{}=(\alpha _2S - \eta _2I_{12}-\gamma _2I_1- \mu _2)I_2 \\ I_{12}' &{}=(\alpha _3S+ \eta _1I_1+\eta _2I_2-\mu _3)I_{12}+\overline{\gamma }I_1I_2 \end{array} \right. \end{aligned}$$

Furthermore, we only consider the case when the reproduction rate of the susceptible class is not less than their death rate, i.e.

$$\begin{aligned} r>0 \quad \Leftrightarrow \quad b>d_0. \end{aligned}$$

Indeed, it is easy to see that the population will go extinct otherwise. The reduced system is considered under the natural initial conditions

4 $$\begin{aligned} S(0)>0,\quad I_1(0)\ge 0, \quad I_2(0)\ge 0,\quad I_{12}(0)\ge 0. \end{aligned}$$

Then it easily follows that any integral curve of (1) with (4) is well-defined and staying in the non negative cone for all $t\ge 0$.

### Reproduction rates

It is convenient to introduce the notation

$$\begin{aligned} \sigma _i:=\frac{\mu _i}{\alpha _i}, \qquad 1\le i\le 3. \end{aligned}$$

We shall always assume that the strains 1 and 2 are different, i.e. $\sigma _1 \ne \sigma _2.$ Then by change of the indices (if needed) we may assume that

$$\begin{aligned} \sigma _1 < \sigma _2. \end{aligned}$$

Under this assumption, $I_1$ is the primary disease, by which we mean that it is the disease most inclined to spread through a naive population.

Furthermore, let us first assume that the populations of the susceptible class and *only one* infected class are non-zero. Let us suppose that only $I_i$ (for some fixed $i\in \{1,2\}$) is non-zero. Then (3) reduces to

5 $$\begin{aligned} \left\{ \begin{array}{ll} S' &{}=(r(1-\frac{S}{K})-\alpha _iI_i)S \\ I_i' &{}=\alpha _i(S - \sigma _i)I_i \end{array} \right. \end{aligned}$$

It is easy to see that there always exist two equilibrium points: the trivial equilibrium $E_1=(0,0)$ and the disease-free equilibrium $E_2=(K,0)$. If $K>\sigma _i$ then also exists (in the positive cone) the coexistence equilibrium $E_3=(\sigma _i, \frac{r}{\alpha _i}(1-\frac{\sigma _i}{K}))$. Next, an elementary analysis shows that the following is true.

#### Proposition 1

The trivial equilibrium state $E_1$ is always unstable. For any positive $K\ne \sigma _i$ there exists a unique locally stable equilibrium point *E*(*K*):

- if $$0<K<\sigma _i$$ then $$E(K)=E_2$$;
- if $$K>\sigma _i$$ then $$E(K)=E_3$$.

The reproduction number

6 $$\begin{aligned} R_0(I_i):=\frac{K}{\sigma _i} \end{aligned}$$

can be used as a threshold. In other words, the transition, with increasing *K*, from the disease-free equilibrium state to the disease equilibrium (the coexistence equilibrium point) occurs exactly when the reproduction number $R_0(I_i)$ of the corresponding infected class $I_i$ exceeds 1. We illustrate the transition by the diagram

$$\begin{aligned} E_2 \rightarrow E_3. \end{aligned}$$

The latter also clarifies the meaning of the parameter $\sigma _i$ as the critical carrying capacity. Note that a more aggressive virus *I* has a greater value of $R_0(I)$. For a fixed value of the carrying capacity *K* this implies that a more aggressive virus *I* has a smaller value of $\sigma$ (which, for example, means smaller recovery rate $\rho$ or greater rate of transmission $\alpha$).

It is natural to assume that the reproduction number of coinfection must be less than that of virus 1 and 2 respectively [15]. Due to this fact, it is natural to assume the following:

7 $$\begin{aligned} \sigma _1< \sigma _2 < \sigma _3. \end{aligned}$$

### Some important notation

In order to keep expressions short we will use the following notations

8 $$\begin{aligned} \Delta _\alpha =\left| \begin{array}{cc} \eta _1 &{}\quad \alpha _1 \\ \eta _2 &{}\quad \alpha _2 \\ \end{array} \right| =\eta _1\alpha _2-\eta _2\alpha _1 \end{aligned}$$

and

9 $$\begin{aligned} \Delta _\mu =\left| \begin{array}{cc} \eta _1 &{}\quad \mu _1 \\ \eta _2 &{}\quad \mu _2 \\ \end{array} \right| =\eta _1\mu _2-\eta _2\mu _1. \end{aligned}$$

We shall assume that the parameters of (3) satisfy the following non-degenerate condition:

10 $$\begin{aligned} \Delta _\alpha \ne 0. \end{aligned}$$

This condition has a natural biological explanation: the virus strains 1 and 2 have different (co)infections rates. Let us define

11 $$\begin{aligned} A_1&= \frac{\alpha _1\alpha _3}{r}(\sigma _3-\sigma _1),\qquad \eta _1^*:=\frac{\eta _1}{A_1} \end{aligned}$$

12 $$\begin{aligned} A_2&= \frac{\alpha _2\alpha _3}{r}(\sigma _3-\sigma _2),\qquad \eta _2^*:=\frac{\eta _2}{A_2} \end{aligned}$$

13 $$\begin{aligned} A_3&= \frac{\alpha _1\alpha _2}{r}(\sigma _2-\sigma _1), \qquad \gamma ^*:=\frac{\gamma _1}{A_3}. \end{aligned}$$

By (7) $A_1,A_2,A_3>0$. We also have

14 $$\begin{aligned} \alpha _2A_1=\alpha _3A_3+ \alpha _1A_2 \end{aligned}$$

and

15 $$\begin{aligned} \Delta _\mu =\frac{\eta _1r}{\alpha _1}A_3+\sigma _1\Delta _\alpha = \frac{\eta _2r}{\alpha _2}A_3+\sigma _2\Delta _\alpha , \end{aligned}$$

hence $$A_3>0$$ implies

16 $$\begin{aligned} \Delta _\mu>\sigma _1\Delta _\alpha \qquad \Delta _\mu >\sigma _2\Delta _\alpha . \end{aligned}$$

This implies an inequality which will be useful in the further analysis:

17 $$\begin{aligned} \sigma _2(\Delta _\alpha +\gamma _2\alpha _3)<\Delta _\mu +\gamma _2\mu _3. \end{aligned}$$

We shall further make use of the following relations:

18 $$\begin{aligned} \begin{aligned}\eta _1 A_2 -\eta _2A_1&< \eta _1\frac{\alpha _2}{\alpha _1}A_1-\eta _2A_1=\Delta _\alpha \frac{A_1}{\alpha _1}. \end{aligned} \end{aligned}$$

On the other hand, one has

19 $$\begin{aligned} \eta _2^*- \eta _1^* = \frac{(\Delta _\mu -\Delta _\alpha \sigma _3)\alpha _3}{A_1A_2r} \end{aligned}$$

#### Remark 1

The parameters $\eta _i^*$ can be thought of as the normalized co-infection rates. They play a distinguished role in the analysis of the thresholds given below.

### The carrying capacity

The concise meaning of the parameter *K* becomes clear if we consider the limit case of (3) when the virus infection is absent, i.e. $I_1=I_2=I_{12}=0$. Then (1) reduces to the system

20 $$\begin{aligned} S'= & {} r\left( 1-\frac{S}{K}\right) S \end{aligned}$$

21 $$\begin{aligned} R'= & {} -\mu _4'R, \end{aligned}$$

where the first Eq. (20) is the famous logistic (Verhulst) equation, *r* is the *intrinsic rate of natural increase* and *K* is the *carrying capacity* of the system. The carrying capacity *K* is one of the most fundamental parameters in population dynamics and it usually expresses the upper limit on the size of hypothetical populations, thereby enhancing mathematical stability. In basic ecology one defines carrying capacity as the equilibrium population size. Indeed, coming back to (3), we can see that *K* coincides with the healthy population size for the *disease-free equilibrium*. Mathematically this means that for any positive initial data, the corresponding solution of (20) converges to *K* as $t\rightarrow \infty$. Furthermore, the equilibrium state $G_{000}:=(K, 0,0,0)$ is the only possible equilibrium point of (3) with all $I_i=0$.

### The main result

Equilibrium points of (3) are determined by the system

22 $$\begin{aligned} \begin{aligned} \left( r\left( 1-\frac{S}{K}\right) -\alpha _1I_1-\alpha _2I_2-\alpha _3I_{12}\right) S=0,\\ (\alpha _1S - \eta _1I_{12}-\gamma _1I_2 - \mu _1)I_1=0,\\ (\alpha _2S - \eta _2I_{12}-\gamma _2I_1- \mu _2)I_2=0,\\ (\alpha _3S+ \eta _1I_1+\eta _2I_2-\mu _3)I_{12}+\overline{\gamma }I_1I_2=0. \end{aligned} \end{aligned}$$

It is elementary to see (see also Proposition 3 below for more explicit representations) that except for the trivial equilibrium point

$$\begin{aligned} {O}=(0,0,0,0) \end{aligned}$$

and the disease-free equilibrium

$$\begin{aligned} G_{000}=(K, 0,0,0), \end{aligned}$$

there exist only 6 possible equilibrium points. The indices $i,j,k\in \{0,1\}$, in the notation $G_{i,j,k}$ are boolean variables that indicates if the corresponding disease compartment is nonzerp or not.

- three semi-trivial equilibria $G_{100},G_{010},G_{001}$ with *only one nonzero infected class*, i.e. $I_{i}\ne 0$ for some *i*;
- two *coinfected semi-trivial equilibria* $G_{101},G_{011}$ with $I_{12}\ne 0$ but $I_1I_2=0$;
- the *coexistence equilibrium* $G_{111}$ with $SI_1I_2I_{12}\ne 0$.

Our main result extends the results obtained in [12] on the case of arbitrary values of $\gamma _i$. More precisely, we will prove that we have the following possible scenarios for developing of an equilibrium point as a continuous function of increasing carrying capacity *K*:

#### Theorem 1

Let us assume that

23 $$\begin{aligned} 0<\eta _1^*< \max \{1, \eta _2^*\}. \end{aligned}$$

Then there is exactly one locally stable nonnegative equilibrium point. Furthermore, changing the carrying capacity *K*, the type of this locally stable equilibrium point may be exactly one of the following alternative cases:

(i) for $\eta _1^*< 1$ one has $G_{000}\rightarrow G_{100}$. More precisely,
  if $0< K<\sigma _1$ then $G_{000}$ is locally stable;
  if $K>\sigma _1$ then $G_{100}$ is locally stable.
(ii) for $1<\eta _1^*<\eta _2^*$ one has $G_{000}\rightarrow G_{100}\rightarrow G_{101}\rightarrow G_{001}$. More precisely,
  if $0< K< \sigma _1$ then $G_{000}$ is locally stable;
  if $\sigma _1<K< K_1$ then $G_{100}$ is locally stable;
  if $K_1<K< K_2$ the point $G_{101}$ is locally stable;
  if $K> K_3$ then the point $G_{001}$ is locally stable
  where
  $$\begin{aligned} K_1=\frac{\sigma _1\eta _1^*}{\eta _1^*-1},\qquad K_2=\frac{\sigma _3}{\sigma _1}K_1. \end{aligned}$$

#### Remark 2

We consider the remaining case

$$\begin{aligned} \eta _1^*> \min \{1, \eta _2^*\} \end{aligned}$$

in the forthcoming paper [3]. This requires a delicate bifurcation analysis with application of methods similar to the principle of the exchange of stability developed in [8]; see also [9] and [5] for recent applications in population analysis. We will show that in the remained cases one has the following two transition diagrams:

(iii) $$G_{000} \rightarrow G_{100}\rightarrow G_{101}\rightarrow G_{111}\rightarrow G_{011}\rightarrow G_{001}$$;
(iv) $$G_{000}\rightarrow G_{100}\rightarrow G_{101}\rightarrow G_{111}$$.

Furthermore, $G_{111}$ may loose stability for large *K* and small $\gamma _i$ in the latter case.

#### Remark 3

In particular, the above result implies that there are only three possible ‘final destination’ equilibrium states, namely $G_{100}, G_{001}$ and $G_{111}$. These are thus the possible scenarios for high density populations where the disease can spread easily due to crowdedness.

## Basic properties of equilibrium points

First we discuss some general results and equilibrium point analysis for (1).

### A priori bounds

In this section we discuss only stable equilibrium points with nonnegative coordinates. We denote

$$\begin{aligned} Y=(S, I_1,I_2,I_{12}). \end{aligned}$$

In what follows, by an *equilibrium point* we always mean an equilibrium *Y* of (3) with nonnegative coordinates, $Y=(S, I_1,I_2,I_{12})\ge 0$.

In the next sections we identify all equilibria of the system (3) and determine their local stability properties. First, let us remark some useful relations which hold for *any* nonnegative equilibrium point of (3).

#### Lemma 1

Let $Y=(S,I_1,I_2,I_{12})\ne (0,0,0,0)$ be a nontrivial equilibrium point of (3) with nonnegative coordinates. Then

24 $$\begin{aligned} 0<S\le K, \end{aligned}$$

and the right equality holds if and only if $I_1=I_2=I_{12}=0$, i.e. precisely when

$$\begin{aligned} Y=G_{000}:=(K,0,0,0). \end{aligned}$$

Furthermore,

25 $$\begin{aligned} \sigma _1\le S\le \min \{K,\sigma _3\}, \end{aligned}$$

unless $$Y=G_{000}$$.

#### Proof

Let $S=0$. Then we have from the second equation of (22) that $(\eta _1I_{12}+\gamma _1I_2 + \mu _1)I_1=0$, where the nonnegativity assumption gives $\eta _1I_{12}+\gamma _1I_2 + \mu _1\ge \mu _1>0$, hence $I_1=0$. For the same reason, $I_2=0$, thus the last equation in (22) yields $\mu _3I_{12}=0,$ hence $I_{12}=0$ too. This proves that $Y=(0,0,0,0)$, hence implying the left inequality in (24).

Now assume that $Y=(S,I_1,I_2,I_{12})\ne (0,0,0,0)$ is an equilibrium point. Since $S\ne 0$, we have from the first equation of (22) that

26 $$\begin{aligned} \alpha _1I_1+\alpha _2I_2+\alpha _3I_{12}=\frac{r(K-S)}{K}. \end{aligned}$$

In particular, the nonnegativity of the left hand side in the latter identity implies that $K-S\ge 0$, i.e. proving the right inequality in (24). On the other hand, summing up the equations in (22) we obtain

27 $$\begin{aligned} \mu _1I_1+\mu _2I_2+\mu _3I_{12}=\frac{r(K-S)S}{K}. \end{aligned}$$

Assuming that $S\ne K$ and dividing (27) by (26) we get

$$\begin{aligned} S=\frac{\mu _1I_1+\mu _2I_2+\mu _3I_{12}}{\alpha _1I_1+\alpha _2I_2+\alpha _3I_{12}} \end{aligned}$$

which readily yields (25). $\square$

This implies, in particular

#### Corollary 1

For any equilibrium point $Y\ne (0,0,0,0)$ and $Y\ne G_{000}$ there holds $K\ge \sigma _1$.

Notice that for $G_{000}$, all $I_i=0$, otherwise we have

#### Corollary 2

If an equilibrium point *Y* is distinct from $G_{000}:=(K,0,0,0)$ then (26) implies the following a priori bound on the *I*-coordinates:

28 $$\begin{aligned} \sigma _1\le S\le \sigma _3, \qquad 0\le I_i\le \frac{r}{\alpha _i}, \qquad i=1,2,3, \end{aligned}$$

where *r* is the intrinsic rate of natural increase. In other words, any equilibrium point distinct from $G_{000}$ lies inside a block with sides depending only on the fundamental constants.

The trivial equilibrium point ${O}=(0,0,0,0)$ is the equilibrium of no disease or susceptible and the standard (local asymptotic) stability treatment shows that this point is always unstable. The first nontrivial equilibrium point $G_{000}$ is the *disease-free equilibrium*, i.e

$$\begin{aligned} G_{000}=(K, 0,0,0) \end{aligned}$$

and it always exist (for any admissible values of the fundamental parameters). The argument of [12] is also applicable in the present case because the stability analysis for $G_{000}$ does not involve $\gamma _i$, so it is literally equivalent to that given in [12]. Repeating this argument (see section 8 in [12]) readily yields the following criterium.

#### Proposition 2

The following three conditions are equivalent:

- the disease-free equilibrium point $G_{000}$ is locally stable;
- the disease-free equilibrium point $G_{000}$ is globally (asymptotically) stable;
- $$0< K<\sigma _1.$$

#### Remark 4

The latter proposition is completely consistent with the dichotomy of the $R_0$-number (the reproduction number, sometimes called basic reproductive ratio). Recall that in epidemiology, the basic reproduction number of an infection is the expected number of cases directly generated by one case in a population where all individuals are susceptible to infection. In our case, using the formal definition (see for example [10]), one has

$$\begin{aligned} R_0=\max \left\{ \frac{K}{\sigma _i}:1\le i\le 3\right\} =\frac{K}{\sigma _1}, \end{aligned}$$

using the fact that the first strain is the most inclined to spread.

In this notation, $R_0<1$ corresponds exactly to the scenario when the infection will die out in the long run (i.e. the only asymptotically stable equilibrium state is the disease-free equilibrium point $G_{000}$), while $R_0>1$ means the infection will be able to spread in a population. Therefore, in what follows, we shall focus on the nontrivial case $R_0>1$ with different scenario admitting the equilibrium states with some of $I_1,I_2,I_{12}$ nonzero.

### Explicit representations of equilibrium points

Coming back to (22), note that the Bezout theorem yields (in generic setting) that a quadratic system with four equations and four independent variables has $2^{4}=16$ distinct solutions (counting the identically zero solution (0, 0, 0, 0)). In fact, in our case we have only one-half of the relevant (the Bezout number) solutions. More precisely, we have

#### Proposition 3

Except for the trivial equilibrium ${O}=(0,0,0,0)$ and the disease-free equilibrium $G_{000}=(K, 0,0,0)$ there exist only the following equilibrium states:

29 $$\begin{aligned} G_{100}&=\left( \sigma _1, I_1, 0,0\right) ,\quad I_1 =\frac{r}{\alpha _1}\left( 1- \frac{\sigma _1}{K}\right) , \end{aligned}$$

30 $$\begin{aligned} G_{010}&=(\sigma _2,0,I_2, 0),\quad I_2:=\frac{r}{\alpha _2}\left( 1- \frac{\sigma _2}{K}\right) , \end{aligned}$$

31 $$\begin{aligned} G_{001}&=(\sigma _3,0,0,I_{12})\quad I_{12}=\frac{r}{\alpha _2}\left( 1- \frac{\sigma _3}{K}\right) , \end{aligned}$$

32 $$\begin{aligned} G_{101}&=(S,I_1,0,I_{12}), \,\,\, S=\frac{\sigma _1K}{K_1}, \,\,\, I_1=\frac{\mu _3}{\eta _1}\left( 1-\frac{K}{K_2}\right) ,\,\,\, I_{12}=\frac{\mu _1}{\eta _1}\left( \frac{K}{K_1}-1\right) , \end{aligned}$$

33 $$\begin{aligned} G_{011}&=(S,0,I_2,I_{12}), \,\,\, S=\frac{\sigma _1K}{K_3},\,\, I_2=\frac{\mu _3}{\eta _2}\left( 1-\frac{K}{K_4}\right) ,\,\,\, I_{12}=\frac{\mu _2}{\eta _2}\left( \frac{K}{K_3}-1\right) , \end{aligned}$$

34 $$\begin{aligned} G_{111}&=(S,I_1,I_2,I_{12}), \end{aligned}$$

where

$$\begin{aligned} K_3=\frac{\sigma _2\eta _2^*}{\eta _2^*-1},\qquad K_4=\frac{\sigma _3}{\sigma _2}K_3. \end{aligned}$$

and there may exist at most two distinct points of type $G_{111}$.

#### Proof

Let $Y=(S,I_1,I_2,I_{12})\ne {O}, G_{000}$ be an equilibrium point. Then by Lemma 1$S>0$ and by the assumption some of coordinates $I_1,I_2,I_{12}$ must be distinct from zero. First assume that $I_{12}=0$. Then the last equation in (22) implies $I_1I_2=0$. By the made assumption this implies that exactly one of $I_1$ and $I_2$ is nonzero while another vanishes. This yields $G_{100}$ and $G_{010}$ in (29) and (41), respectively. Now, let $I_{12}\ne 0$ but $I_1I_2=0$. Then the last equation in (22) implies $\alpha _3S+ \eta _1I_1+\eta _2I_2-\mu _3=0$. An elementary analysis reveals exactly three possible points $G_{001}, G_{101}$ and $G_{011}$ in (31)–(33). Finally, consider the case when all coordinates of *Y* are distinct from zero. Since *Y* is distinct from *O* and $G_{000}$, it must satisfy (26), (27). Also, since $I_1,I_2\ne 0$, we obtain from the second and the third equations (22) the following system:

$$\begin{aligned} \mu _1I_1+\mu _2I_2+\mu _3I_{12}&=\frac{r}{K}(K-S)S,\\ \alpha _1I_1+\alpha _2I_2+\alpha _3I_{12}&=\frac{r}{K}(K-S),\\ \alpha _1S-\gamma _1I_2 - \eta _1I_{12} - \mu _1&=0,\\ \alpha _2S-\gamma _2I_1 - \eta _2I_{12}- \mu _2&=0. \end{aligned}$$

Rewriting these four equations in the matrix form as follows

35 $$\begin{aligned} \left( \begin{matrix} \mu _1 &{}\quad \mu _2 &{}\quad \mu _3 &{}\quad \frac{r}{K}(S-K)S \\ \alpha _1 &{}\quad \alpha _2 &{}\quad \alpha _3 &{}\quad \frac{r}{K}(S-K) \\ 0 &{}\quad \gamma _1 &{}\quad \eta _1 &{}\quad \mu _1-\alpha _1S \\ \gamma _2 &{}\quad 0 &{}\quad \eta _2 &{}\quad \mu _2-\alpha _2S \\ \end{matrix} \right) \left( \begin{matrix} I_1 \\ I_2 \\ I_{12}\\ 1\\ \end{matrix} \right) = \left( \begin{matrix} 0 \\ 0 \\ 0\\ 0\\ \end{matrix} \right) \end{aligned}$$

we conclude that $(I_1,I_2,I_{12},1)^T$ is a 0-eigenvector of the matrix in the left hand side of (35), thus, the first coordinate *S* satisfies the determinant equation

$$\begin{aligned} P(S):=p_2S^2+p_1S+p_0=0, \end{aligned}$$

where

36 $$\begin{aligned} P(S):= \begin{vmatrix} \mu _1&\quad \mu _2&\quad \mu _3&\quad \frac{r}{K}(S-K)S \\ \alpha _1&\quad \alpha _2&\quad \alpha _3&\quad \frac{r}{K}(S-K) \\ 0&\quad \gamma _1&\quad \eta _1&\quad \mu _1-\alpha _1S \\ \gamma _2&\quad 0&\quad \eta _2&\quad \mu _2-\alpha _2S \\ \end{vmatrix} \end{aligned}$$

and

$$\begin{aligned} p_0= & {} \begin{vmatrix} \mu _1&\quad \mu _2&\quad \mu _3&\quad 0 \\ \alpha _1&\quad \alpha _2&\quad \alpha _3&\quad \mu _0-b \\ 0&\quad \gamma _1&\quad \eta _1&\quad \mu _1 \\ \gamma _2&\quad 0&\quad \eta _2&\quad \mu _2 \\ \end{vmatrix} ,\quad p_1=\begin{vmatrix} \mu _1&\quad \mu _2&\quad \mu _3&\quad \mu _0-b \\ \alpha _1&\quad \alpha _2&\quad \alpha _3&\quad \frac{r}{K} \\ 0&\quad \gamma _1&\quad \eta _1&\quad -\alpha _1 \\ \gamma _2&\quad 0&\quad \eta _2&\quad -\alpha _2 \\ \end{vmatrix} ,\quad \\ p_2= & {} \begin{vmatrix} \mu _1&\quad \mu _2&\quad \mu _3&\quad \frac{r}{K} \\ \alpha _1&\quad \alpha _2&\quad \alpha _3&\quad 0 \\ 0&\quad \gamma _1&\quad \eta _1&\quad 0 \\ \gamma _2&\quad 0&\quad \eta _2&\quad 0 \\ \end{vmatrix} \end{aligned}$$

In particular, it follows that *P*(*S*) is a quadratic polynomial in *S*, therefore there may be at most two distinct inner points of type $G_{111}$. The condition $P(S)=0$ is sufficient if $\gamma _1,\gamma _2<\frac{\Delta _\alpha }{\alpha _3}$. $\square$

It follows from Proposition 3 that all the boundary (edge) stationary points are uniquely determined and can be found by explicit formulas. The existence and uniqueness of coexistence (inner) points of type $G_{111}$ is more involved (in contrast with the Lotka-Volterra case ${\bar{\gamma }}=0$) and depends on the value of ${\bar{\gamma }}$.

We study the existence and the local stability of inner points by a bifurcation approach in the forthcoming paper [3]. Notice also that in the particular case $\gamma _i=0$, the characteristic polynomial (36) becomes a linear function expressed explicitly by

$$\begin{aligned} P(S)|_{\gamma _1=\gamma _2=0}&= \alpha _1\alpha _2(\sigma _1-\sigma _2)(\Delta _\mu -S\Delta _\alpha ) \end{aligned}$$

where we used the notation in (15). This considerably simplifies the analysis, see [12].

#### Lemma 2

The following holds:

(i) For each $G_j$, $j=1,2,3,5$, there exists $\varepsilon >0$ (depending on the fundamental parameters $\alpha _i, \mu _i$, $\eta _i$ and $\gamma _i$) such that $\Vert G_j-G_{111}\Vert \ge \varepsilon$.
(ii) Let $G_{010}$ be given by (30) and $\delta :=\alpha _1S^*-\gamma _1I_2^*-\mu _1> 0$ (or equivalently $\gamma ^*<K/(K-\sigma _2)$). Then there exists $\varepsilon (\delta )>0$ such that $\Vert G_{010}-G_{111}\Vert \ge \varepsilon (\delta )$.
(iii) Let $G_{101}$ be given by (32) and $\delta :=\alpha _2S^*-\eta _2I_{12}-\gamma _2I_1^*-\mu _2\ne 0$. Then there exists $\varepsilon (\delta )>0$ such that $\Vert G_{101}-G_{111}\Vert \ge \varepsilon (\delta )$.
(iv) Let $G_{011}$ be given by (34) and $\delta :=\alpha _1S^*-\eta _1I_{12}-\gamma _1I_1^*-\mu _1\ne 0$. Then there exists $\varepsilon (\delta )>0$ such that $\Vert G_{011}-G_{111}\Vert \ge \varepsilon (\delta )$.

#### Proof

(i) We prove the assertion for $j=5$ since the other cases are considered in a similar way. The second and the third equations in (22) near the point $G_{001}$ have the form

37 $$\begin{aligned} (\alpha _1K-\mu _1+O(\epsilon ))I_1=0,\;\;(\alpha _2K-\mu _2+O(\epsilon ))I_2=0, \end{aligned}$$

where $\epsilon =\Vert G_{001}-G_{111}\Vert$. By the assumption (7), one of the numbers $\alpha _1K-\mu _1$, $\alpha _2K-\mu _2$ does not vanish and so the corresponding coefficient in (37) does not vanish for small $\epsilon$, which implies (i) for $G_{001}$. Proofs of (ii)–(iv) use the same argument.

$$\square $$

### Equilibrium branches

It turns out that the most natural way to study equilibrium points is to consider their dependence on the carrying capacity *K* . We know by Proposition 2 that the disease-free equilibrium point $G_{000}$ is the only stable equilibrium point for $0\le K< \sigma _1$. In this section we consider each equilibrium state separately and study their local stability for $K\ge \sigma _1$. We study first the local stability of each point individually and in the next sections consider the dependence on *K*.

Our main goal is to describe all possible continuous scenarios of how the locally stable equilibrium states of (3) depends on *K* provided that all other fundamental parameters $\alpha _i$, $\mu _i$, *b*, $\gamma _i$ remain fixed. To this end, we introduce the following concept.

#### Definition 1

By an *equilibrium branch* we mean any continuous in $K\ge 0$ family of equilibrium points of (3) which are locally stable for all but finitely many threshold values of *K*.

#### Remark 5

We need to distinguish the threshold values of *K* in the above definition because, formally, the local stability (i.e. that the real parts of all the systems characteristic roots are negative) fails when an equilibrium point changes its type. On the other hand, a branch may be stable in the Lyapunoff sense even for the threshold values of *K*. Indeed, the latter holds at least for $\gamma =0$, see [12].

## The equilibrium state $G_{100}$: Proof of (i)

Note that the next three boundary equilibriums $G_{100},G_{010}$ and $G_{001}$ have a constant *S*-coordinate (independent on *K*). The first of these is the equilibrium point $G_{100}$ with the presence of only the first strain. Its explicit expression with the nonnegativity condition are given by (29). Remark that when $K= \sigma _1$, the globally stable equilibrium point $G_{000}$ bifurcates into $G_{100}=(\sigma _1, I_1^*, 0,0)$:

$$\begin{aligned} G_{100} = G_{000} \quad \text { when }\quad I_1^* = 0 \Leftrightarrow K= \sigma _1 \end{aligned}$$

Using (29), we find the corresponding Jacobian matrix evaluated at $G_{100}$:

$$\begin{aligned} J_{100}=\begin{bmatrix} -\frac{r\sigma _1}{K} &{} -\alpha _1\sigma _1 &{} -\alpha _2\sigma _1 &{} -\alpha _3\sigma _1 \\ \alpha _1I_1^* &{} 0 &{} -\gamma _1I_1^* &{} -\eta _1I_1^* \\ 0 &{} 0 &{} -\alpha _2(\sigma _2-\sigma _1)-\gamma _2I_1^* &{} 0 \\ 0 &{} 0 &{} \overline{\gamma }I_1^* &{} -\alpha _3(\sigma _3-\sigma _1)+\eta _1I_1^* \end{bmatrix}, \end{aligned}$$

Notice that, $J_{100}$ has a block structure. The left upper $2\times 2$-block is obviously stable. Therefore $J_{100}$ is stable if and only if the right lower block is so. By virtue of $-\alpha _2(\sigma _2-\sigma _1)-\gamma _2I_1^*<0$ this is equivalent to

38 $$\begin{aligned} -\alpha _3(\sigma _3-\sigma _1)+\eta _1I_1^* <0, \end{aligned}$$

or, equivalently, using the expression $I_1^* =\frac{r}{K \alpha _1}(K-\sigma _1)$ and (11) we obtain

39 $$\begin{aligned} \eta _1^*< \frac{K}{K-\sigma _1}. \end{aligned}$$

After some obvious manipulations we arrive at

### Proposition 4

The equilibrium point $G_{100}$ is stable nonnegative if and only if

40 $$\begin{aligned} \left\{ \begin{array}{cc} K>\sigma _1&{}\quad \text {if}\quad \eta _1^*\le 1\\ &{}\\ \sigma _1<K<K_1&{} \quad \text {if}\quad \eta ^*_1>1. \end{array} \right. \end{aligned}$$

Notice that the point $G_{100}$ remains nonnegative and locally stable for *any* $K>\sigma _1$ provided $\eta ^*_1\le 1$. This provides us with the first (simplest) example of a branch. More precisely, we have

### Corollary 3

(Branch (i)) Let $\eta ^*_1\le 1$. Then

- for $0< K<\sigma _1$ the point $G_{000}$ is locally (in fact, globally) stable;
- for $K=\sigma _1$ the point $G_{000}$ coincides with $G_{100}$;
- for $K>\sigma _1$ the point $G_{100}$ is locally stable.

We display this schematically as

$$\begin{aligned} G_{000}\rightarrow G_{100}. \end{aligned}$$

The latter corollary implies (i) in Theorem 1.

## Proof of (ii)

Corollary 3 completely describes all possible scenarios for $0\le K<\infty$ when $\eta ^*_1\le 1$. In what follows, we shall always assume that $\eta ^*_1>1$. Then Proposition 4 tells us that $G_{100}$ remains locally stable for any $\sigma _1<K<K_1$. If we want to find a *continuous* equilibrium branch, we need to check which of the remained candidates $G_{010},G_{001},G_{101},G_{011},G_{111}$ becomes equal to $G_{100}$ for the right critical value $K=K_1$.

An easy inspection shows that for a generic choice of the fundamental parameters there is only one possible candidate, namely $G_{101}$. Thus, to construct the only possible scenario for a continuous equilibrium branch is when $G_{100}$ bifurcates into $G_{101}$. In the next section we give stability analysis of $G_{010}$ and $G_{001}$, and then continue with $G_{101}$ and construction of equilibrium branches.

### The equilibrium state $G_{010}$

The equilibrium point $G_{010}$ expresses the presence of only the second strain, see (30). It is nonnegative if and only if

41 $$\begin{aligned} K>\sigma _2 . \end{aligned}$$

Note that if $G_{010}$ is nonnegative then by virtue of (41) and (7), $G_{100}$ is nonnegative too. The Jacobian matrix computed at $G_{010}$ is

42 $$\begin{aligned} J_{010}= \begin{bmatrix} -r\frac{\sigma _2}{K} &{}\quad -\alpha _1\sigma _2 &{}\quad -\alpha _2\sigma _2 &{}\quad -\alpha _3\sigma _2 \\ 0 &{}\quad \alpha _1(\sigma _2-\sigma _1)-\gamma _1I_2^* &{}\quad 0 &{}\quad 0 \\ \alpha _2I_2^* &{}\quad -\gamma _2I_2^* &{}\quad 0 &{}\quad -\eta _2I_2^* \\ 0 &{}\quad \overline{\gamma }I_2^* &{}\quad 0 &{}\quad -\alpha _3(\sigma _3-\sigma _2)+\eta _2I_2^* \end{bmatrix} \end{aligned}$$

Note that, interchanging rows and columns of the matrix (42) only change the sign of the determinant of this matrix. Therefore, after an obvious rearrangement, the eigenvalues of $J_{010}$ solves the following equation:

43 $$\begin{aligned} \begin{vmatrix} -r\frac{\sigma _2}{K}-\lambda&\quad -\alpha _2\sigma _2&\quad -\alpha _1\sigma _2&\quad -\alpha _3\sigma _2 \\ \alpha _2I_2^*&\quad -\lambda&\quad -\gamma _2I_2^*&\quad -\eta _2I_2^* \\ 0&\quad 0&\quad \alpha _1(\sigma _2-\sigma _1)-\gamma _1I_2^* -\lambda&\quad 0 \\ 0&\quad 0&\quad \overline{\gamma }I_2^*&\quad -\alpha _3(\sigma _3-\sigma _2)+\eta _2I_2^*-\lambda \end{vmatrix}=0.\nonumber \\ \end{aligned}$$

Again, one easily verifies that the left upper $2\times 2$-block is stable, while the stability of the right down (lower-diagonal) block is equivalent to the negativity of the diagonal elements, i.e. to the inequalities

$$\begin{aligned} \left\{ \begin{array}{rl} \alpha _1(\sigma _2-\sigma _1)-\gamma _1I_2^* &{}<0,\\ -\alpha _3(\sigma _3-\sigma _2)+\eta _2I_2^*&{}<0. \end{array} \right. \end{aligned}$$

Thus the stability of $G_{010}$ is equivalent to the inequalities

44 $$\begin{aligned} \left\{ \begin{array}{rl} K\left( 1-\frac{1}{\gamma ^*}\right) >\sigma _2\\ K<K_3, \end{array} \right. \end{aligned}$$

where $\gamma ^*:=\frac{\gamma _1}{A_3}$. In summary, we have

#### Proposition 5

The equilibrium point $G_{010}$ is stable and nonnegative iff

- $K_3<K< \frac{\sigma _2\gamma ^*}{\gamma ^*-1}$ when $\gamma ^*>1$ and $\eta _2^*>1$, or
- $$K>\frac{\sigma _2\gamma ^*}{\gamma ^*-1}$$ when $$\gamma ^*>1$$ and $$\eta _2^*<1$$.

#### Remark 6

In this paper, we are primarily interested in the case of ‘small’ values of $\gamma _i$. On the other hand, the latter proposition shows that $G_{010}$ may be stable only if $\gamma _1>A_3$, therefore this equilibrium is not stable for small values of $\gamma _1$ and will be eliminated from the subsequent analysis.

#### Corollary 4

The equilibrium point $G_{010}$ is locally unstable if $0\le \gamma _1^*<1$.

### The equilibrium state $G_{001}$

An equilibrium point in the presence of coinfection is given by (31).

#### Proposition 6

The equilibrium point $G_{001}$ is stable and nonnegative iff

45 $$\begin{aligned} \eta :=\min \{\eta _1^*, \eta _2^*\}>1 \quad \text {and}\quad K>\frac{\sigma _3\eta }{\eta -1}. \end{aligned}$$

Furthermore, if the point $G_{001}$ is nonnegative and locally stable for a certain $K_0>0$ then it will be so for any $K\ge K_0$ (provided that other parameters are fixed).

#### Proof

By (31), $I_{12}^*=\frac{r}{K \alpha _3}(K-\sigma _3)$, hence the positivity of $I_{12}^*$ is equivalent to

$$\begin{aligned} K> \sigma _3. \end{aligned}$$

Next, the Jacobian matrix evaluated at $G_{001}$ is

46 $$\begin{aligned} J_{001}= \begin{bmatrix} -r\frac{\sigma _3}{K} &{} -\alpha _1\sigma _3 &{} -\alpha _2\sigma _3 &{} -\alpha _3\sigma _3 \\ 0 &{} \alpha _1(\sigma _3-\sigma _1)-\eta _1I_{12}^* &{} 0 &{} 0 \\ 0 &{} 0 &{} \alpha _2(\sigma _3-\sigma _2)-\eta _2I_{12}^* &{} 0 \\ \alpha _3I_{12}^* &{} \eta _1I_{12}^* &{} \eta _2I_{12}^* &{} 0 \end{bmatrix}, \end{aligned}$$

The matrix has a block structure where the block

$$\begin{aligned} \begin{bmatrix} -r\frac{\sigma _3}{K} &{}\quad -\alpha _3\sigma _3 \\ \alpha _3I_{12}^* &{}\quad 0 \end{bmatrix} \end{aligned}$$

is obviously stable, therefore the stability of $J_{001}$ is equivalent to the negativity of two diagonal elements:

$$\begin{aligned} \alpha _1(\sigma _3-\sigma _1)-\eta _1I_{12}^*<0,\\ \alpha _2(\sigma _3-\sigma _2)-\eta _2I_{12}^*<0. \end{aligned}$$

First notice that stability of $G_{001}$ implies immediately that $I^*_{12}>0$. Also, taking into account that $I_{12}^*=\frac{r}{K \alpha _3}(K-\sigma _3)$, the stability of $G_{001}$ is equivalent to the inequalities

$$\begin{aligned} \sigma _3< K\left( 1-\min \left\{ \frac{1}{\eta ^*_1},\, \frac{1}{\eta ^*_2}\right\} \right) =K\left( 1-\frac{1}{\eta }\right) . \end{aligned}$$

In summary, we have (31). Finally, the last statement of the proposition follows immediately from the increasing (with respect to *K*) character of the second inequality in (45). $\square$

#### Remark 7

We emphasize that the stability of the equilibrium states $G_{000},G_{100},G_{010}$ and $G_{001}$ does not involve the interference parameters $\gamma _1, \gamma _2$.

### The equilibrium state $G_{101}$

Analysis of the remaining three equilibrium points $G_{101},G_{011}$ and $G_{111}$ is more delicate and now also involves the coinfection constants $\gamma _1,\gamma _2.$ Let us consider the boundary equilibrium point

$$\begin{aligned} G_{101}=\left( \frac{\sigma _1K}{K_1}, \,\, \frac{\mu _3}{\eta _1}\left( 1-\frac{K}{K_2}\right) ,\,\,0,\,\, \frac{\mu _1}{\eta _1}\left( \frac{K}{K_1}-1\right) \right) , \end{aligned}$$

see (32). First notice that the coordinates of $G_{101}$ are nonnegative if and only if the two conditions hold: $K_1>0$ (which is equivalent to $\eta ^*_1>1$) and also

$$\begin{aligned} \sigma _1<S^*<\sigma _3. \end{aligned}$$

We see that $G_{101}$ is nonnegative if and only if

47 $$\begin{aligned} K_1<K<K_2, \quad \eta ^*_1>1. \end{aligned}$$

(Note that the bilateral inequality is inconsistent with (45)).

Now let us study the local stability of $G_{101}$. Using (32), the Jacobian matrix for $G_{101}$ is found as

$$\begin{aligned} J_{101}= \begin{bmatrix} -r\frac{S^*}{K } &{}\quad -\alpha _1S^* &{}\quad -\alpha _2S ^*&{}\quad -\alpha _3Sj \\ \alpha _1I_1^* &{}\quad 0 &{}\quad -\gamma _1I_1^* &{}\quad -\eta _1I_1^* \\ 0 &{}\quad 0 &{}\quad \alpha _2S^*-\eta _2I_{12}^*-\gamma _2I_1^*-\mu _2 &{}\quad 0 \\ \alpha _3I_{12}^* &{}\quad \eta _1I_{12}^* &{}\quad \eta _2I_{12}^*+\overline{\gamma }I_1^* &{}\quad 0 \end{bmatrix}. \end{aligned}$$

with $S^*,I^*_1,I^*_{12}$ given by (32). Using the block structure of $J_{101}$, we obtain that $G_{101}$ is locally stable if and only if

- there holds
  48 $$\begin{aligned} \alpha _2S^*-\eta _2I_{12}^*-\gamma _2I_1^*-\mu _2 <0 \end{aligned}$$
- and the matrix below is stable:
  49 $$\begin{aligned} {\tilde{J}}= \begin{bmatrix} -r\frac{S^*}{K} &{}\quad -\alpha _1S ^*&{}\quad -\alpha _3S ^* \\ \alpha _1I_1 ^*&{}\quad 0 &{}\quad -\eta _1I_1^* \\ \alpha _3I_{12}^* &{}\quad \eta _1I_{12}^* &{}\quad 0 \end{bmatrix} = \begin{bmatrix} S^* &{}\quad 0 &{}\quad 0 \\ 0 &{}\quad I_1^* &{}\quad 0 \\ 0 &{}\quad 0 &{}\quad I_{12}^* \end{bmatrix} \begin{bmatrix} -\frac{r}{K} &{}\quad -\alpha _1 &{}\quad -\alpha _3 \\ \alpha _1 &{}\quad 0 &{}\quad -\eta _1 \\ \alpha _3 &{}\quad \eta _1 &{}\quad 0 \end{bmatrix}.\nonumber \\ \end{aligned}$$

The stability of ${\tilde{J}}$ is equivalent to the stability of the last matrix factor in (49). An easy application of the Routh-Hurwitz criteria [11] confirms that ${\tilde{J}}$ is always stable. Hence, the stability of $G_{101}$ is equivalent to the condition (48). Using (32), we can rewrite it as follows:

50 $$\begin{aligned} S^*(\Delta _\alpha +\gamma _2\alpha _3)<\Delta _\mu +\gamma _2\mu _3 \end{aligned}$$

see (8) and (9). Let us define

51 $$\begin{aligned} {\hat{S}}_1:=\frac{\Delta _\mu +\mu _3\gamma _2}{\Delta _\alpha +\alpha _3\gamma _2} \end{aligned}$$

We have by using (11)–(12)

52 $$\begin{aligned} {\hat{S}}_1-\sigma _1&= \frac{\eta _1\alpha _1\alpha _2(\sigma _2-\sigma _2)+ \gamma _2\alpha _1\alpha _3(\sigma _3-\sigma _1)}{\alpha _1(\Delta _\alpha +\alpha _3\gamma _2)}\nonumber \\&=\frac{r(\eta _1A_3+\gamma _2A_1)}{\alpha _1(\Delta _\alpha +\alpha _3\gamma _2)}, \end{aligned}$$

53 $$\begin{aligned} {\hat{S}}_1-\sigma _2&=\frac{\eta _2\alpha _1\alpha _2(\sigma _2-\sigma _1) +\gamma _2\alpha _2\alpha _3(\sigma _3-\sigma _2)}{\alpha _2 (\Delta _\alpha +\alpha _3\gamma _2)}\nonumber \\&=\frac{r(\eta _2A_3+\gamma _2A_2)}{\alpha _1(\Delta _\alpha +\alpha _3\gamma _2)}, \end{aligned}$$

54 $$\begin{aligned} {\hat{S}}_1-\sigma _3&=\frac{\eta _2\alpha _1\alpha _3(\sigma _3-\sigma _1)-\eta _1\alpha _2\alpha _3 (\sigma _2-\sigma _3)}{\alpha _3(\Delta _\alpha +\alpha _3\gamma _2)}\nonumber \\&=\frac{A_1A_2r(\eta _2^*- \eta _1^*)}{\alpha _3(\Delta _\alpha +\alpha _3\gamma _2)}, \end{aligned}$$

Consider first the case $\Delta _\alpha +\alpha _3\gamma _2=0$. Then by (17) it follows that $\Delta _\mu +\gamma _2\mu _3>0$ therefore (50) holds automatically true in this case, and $G_{101}$ is locally stable.

Next consider the case $\Delta _\alpha +\alpha _3\gamma _2<0$. Then it follows from (50) that $G_{101}$ is stable whenever $S^*>{\hat{S}}_1$. On the other hand, (52) implies in this case ${\hat{S}}_1<\sigma _1,$ therefore using (25) we see that

55 $$\begin{aligned} S^*>\sigma _1>{\hat{S}}_1 \end{aligned}$$

whenever $S^*$ is nonnegative. Therefore in this case $G_{101}$ is locally stable whenever (47) are fulfilled. Note also that under the made assumption $\Delta _\alpha +\alpha _3\gamma _2<0$ one necessarily has $\eta _2^*> \eta _1^*$. Indeed, if $\eta _2^*\le \eta _1^*$ then (18) implies $\Delta _\alpha >0$, therefore $\Delta _\alpha +\alpha _3\gamma _2>0$, a contradiction.

Finally, assume that

56 $$\begin{aligned} \Delta _\alpha +\alpha _3\gamma _2>0 \end{aligned}$$

Then by (50) the point $G_{101}$ is locally stable if and only if $S^*<{\hat{S}}_1$, i.e.

57 $$\begin{aligned} K<\frac{{\hat{S}}_1}{1-\frac{1}{\eta _1^*}}. \end{aligned}$$

Under assumption (56), (52) implies ${\hat{S}}_1>\sigma _1$. On the other hand, we have

$$\begin{aligned} {\hat{S}}_1\ge \sigma _3 \text { if } \eta _2^*\ge \eta ^*_1 \quad \text { and }\quad {\hat{S}}_1<\sigma _3 \text { if } \eta _2^*<\eta ^*_1. \end{aligned}$$

On the other hand, in the latter case, the inequality $\eta _2^*\ge \eta ^*_1$ by virtue of (18) that in fact $\Delta _\alpha >0$, therefore (56) holds automatically true in this case. Combining (57) with the nonnegativity condition (47), and summarizing the above observations we arrive at

#### Proposition 7

The equilibrium point $G_{101}$ is nonnegative stable iff $\eta _1^*>1$ and the following conditions hold:

58 $$\begin{aligned} K_1<K<\frac{Q}{\sigma _1}K_1 \end{aligned}$$

where

59 $$\begin{aligned} Q=\left\{ \begin{array}{ll} \sigma _3&{}\quad \text {if }\eta _2^*\ge \eta ^*_1;\\ {\hat{S}}_1&{}\quad \text {if }\eta _2^*<\eta ^*_1. \end{array} \right. \end{aligned}$$

Now we are ready to describe the equilibrium branch for $\eta _1^*>1$.

#### Corollary 5

Let $$\eta _1^*>1$$. Then

- for $0< K<\sigma _1$ the point $G_{000}$ is locally (in fact, globally) stable;
- for $K=\sigma _1$ the point $G_{000}$ coincides with $G_{100}$;
- for $\sigma _1<K<K_1$ the point $G_{100}$ is locally stable;
- for $K=K_1$ the point $G_{100}$ coincides with $G_{101}$;
- for $K_1<K<\frac{Q}{\sigma _1}K_1$ the point $G_{101}$ is locally stable, where *Q* is defined by (59).

We display this schematically as

60 $$\begin{aligned} G_{000}\rightarrow G_{100}\rightarrow G_{101}\rightarrow \ldots \end{aligned}$$

#### Proof

The first three items are obtained by combining Proposition 4 with Proposition 2. Note that the upper bound in (c) here is smaller than that in (c) in Corollary 3. When $K=K_1=\frac{\sigma _1\eta _1^*}{\eta _1^*-1}$, it follows that the $I_{12}$-coordinate of $G_{101}$ vanishes (see (32)), i.e. $G_{101}=G_{100}$, which proves (d). Next, Proposition 7 yields (e). $\square$

With Corollaries 3 and 5 in hand, it is natural to ask: What happens with an equilibrium branch when $\eta ^*_1>1$ and $K>K_1$?

So far, we see that any continuous equilibrium branch develops uniquely determined accordingly (60). But at $G_{101}$ the situation becomes more complicated: this point may a priori bifurcate into different points.

In this paper we only consider the particular case (ii), i.e. when $1<\eta _1^*<\eta _2^*$. This yields by (59) that $Q=\sigma _3$, hence (58) implies that $G_{101}$ is locally stable for

$$\begin{aligned} K_1<K<K_2. \end{aligned}$$

The upper critical value $K_2$ substituted in (32) implies that $I_1^*=0$, hence $G_{101}$ naturally bifurcates into $G_{001}$. It is easy to see that the corresponding $I_{12}^*$ for $G_{001}$ and $G_{101}$ coincide when $K=K_2$ holds. This observation combined with Proposition 6 implies that in this case for any $K>K_2$ the point $G_{001}$ will be locally stable, hence we arrive at

#### Corollary 6

(Branch (ii)) Let $\eta _2^*\ge \eta _1^*>1$ hold. Then

- for $0< K<\sigma _1$ the point $G_{000}$ is locally (in fact, globally) stable;
- for $K=\sigma _1$ the point $G_{000}$ coincides with $G_{100}$;
- for $\sigma _1<K<K_1$ the point $G_{100}$ is locally stable;
- for $K=K_1$ the point $G_{100}$ coincides with $G_{101}$;
- for $K_1<K<K_2$ the point $G_{101}$ is locally stable;
- for $K=K_2$ the point $G_{101}$ coincides with $G_{001}$;
- for $K>K_2$ the point $G_{001}$ is locally stable.

We display this schematically as

61 $$\begin{aligned} G_{000}\rightarrow G_{100}\rightarrow G_{101}\rightarrow G_{001} \end{aligned}$$

### Bifurcation of $G_{101}$

Thus, one remains to study the case when

62 $$\begin{aligned} \eta _2^*< \eta _1^*, \qquad \eta _1^*>1 \end{aligned}$$

hold. Notice that in fact by virtue of (18) the latter inequality implies

63 $$\begin{aligned} \Delta _\alpha >0. \end{aligned}$$

We know by (e) in Corollary 5 that $G_{101}$ is locally stable for

$$\begin{aligned} K_1<K<\frac{{{\hat{S}}}_1\eta _1^*}{\eta _1^*-1}. \end{aligned}$$

Substituting the corresponding critical value $K =K_0$ such that

$$\begin{aligned} K_0=\frac{{{\hat{S}}}_1\eta _1^*}{\eta _1^*-1}= \frac{\Delta _\mu +\mu _3\gamma _2}{\Delta _\alpha +\alpha _3\gamma _2}\cdot \frac{\eta _1^*}{\eta _1^*-1} \end{aligned}$$

in (32) reveals that the coordinates $G_{101}$ *do not vanish*, i.e. $G_{101}$ does not change its type. Instead it losts its local stability because the determinant of $J_{101}$ vanishes at this moment. To continue the equilibrium branch (60) beyond $G_{101}$ we need to find an appropriate candidate for a stable point. By the continuity argument (because $G_{101}$ keeps all coordinates nonzero for $K=K_0$), the only possible candidate for a continuous equilibrium branch is a point of type $G_{111}$. Since we do not have any explicit expression of $G_{111}$, the analysis in this case is more complicated and involves a certain bifurcation technique which we develop in a forthcoming paper [3].

## Concluding remarks

It is natural, from a biological point of view, to relax the constancy condition on the transmission rates $\alpha _i$ and assume that in general they may depend on the carrying capacity. Indeed, a larger carrying capacity can be due to a larger living area for a population in contrast to increased amount of resources in a given area. This would would make a population of given size more sparse. This increased sparseness would make it harder for the strains to spread. With this in mind, one natural assumption is the following relation:

64 $$\begin{aligned} \alpha _i(K)=\frac{a_i}{K}. \end{aligned}$$

This implies for the other fundamental constants

$$\begin{aligned} \sigma _i=\frac{\mu _i}{a_i}K=:s_iK, \end{aligned}$$

and

$$\begin{aligned} A_i=\frac{B_i}{K}, \quad \text {where }\quad B_1=\frac{a_1a_3(s_3-s_1)}{r} \text { etc.} \end{aligned}$$

The main consequence of (64) is that the coordinates of a stable equilibrium point is no longer bounded and develop as *K* increases. For example, under assumption (64) one has from (25) merely

$$\begin{aligned} s_1K\le S\le K\min \{1,s_3\}. \end{aligned}$$

This, in particular implies that already the first bifurcation $S_2\rightarrow S_3$ is completely different. Indeed, it follows from Proposition 2 that $G_{000}$ becomes stable *for all *$K>0$ provided $s_1\ge 1$. In the nontrivial case $s_1<1$, $G_{000}$ is *never* stable. In general, Proposition 4 and Corollary 6 instead imply

### Corollary 7

We have the following stability analysis:

(i) If $s_1\ge 1$ then $G_{000}$ is stable for all $K>0$;
(ii) If $s_1<1$ and $0<\eta _1^*\le \frac{1}{1-s_1}$ then $G_{100}$ stable for all $K>0$;
  Let now $s_1<1$, $\eta _2^*>\eta _1^*> \frac{1}{1-s_1}$ hold. Then
(iii) if $s_3\ge 1$ or $s_3<1$ and $\eta _1^*<\frac{1}{1-s_3}$ then $G_{101}$ stable for all $K>0$;
(iv) if $s_3<1$ and $\eta _1^*>\frac{1}{1-s_3}$ then $G_{001}$ stable for all $K>0$.

Thus, we have a complete description in the cases $\eta _1^*\le 1$ and $\eta _2^*\ge \eta _1^*>1$. The remained case $\eta _1^*\ge \max \{1,\eta _2^*\}$ will be considered in [3].

## Data Availability

The manuscript has no associated data.
